# Complete Mitochondrial Genome and Its Phylogenetic Analysis of *Oides decempunctatus* (Coleoptera: Chrysomelidae)

**DOI:** 10.1002/ece3.71819

**Published:** 2025-07-15

**Authors:** Honghua Zhang, Junhao Wu, Zhiqian Liu, Xinju Wei, Zhihang Zhuo

**Affiliations:** ^1^ College of Life Science China West Normal University Nanchong China

**Keywords:** evolutionary relationships, mitogenome, pest species, protein‐coding genes, ribosomal RNA genes, secondary structure

## Abstract

*Oides decempunctatus* is an insect that parasitizes plants in the grape family and causes significant damage to grape buds and leaves. This study successfully determined the mitochondrial genome sequence of *O. decempunctatus* and analyzed its gene structure and the phylogenetic relationships within the family Chrysomelidae. Using the Illumina HiSeq platform, the complete mitochondrial genome of *O. decempunctatus* was sequenced to examine its genomic structural characteristics and nucleotide composition. Based on the sequences of 13 protein‐coding genes and 2 rRNA genes, along with complete mitochondrial genome sequences of 24 Chrysomelidae species from GenBank, we selected two species from the subfamily Chrysomelinae, *Chrysomela vigintipunctata* and *Chrysomela aeneicollis* , as outgroups. Phylogenetic trees were constructed using maximum likelihood and Bayesian inference methods. The mitochondrial genome of *O. decempunctatus* is 16,062 base pairs long, circular, and double‐stranded, containing 22 tRNA genes, 2 rRNA genes, 13 PCGs, and a control region, with an arrangement monophyletic group. Notably, within the subfamily Chrysomelinae, the species *Gastrolina depressa* and *Gastrolina thoracica* clustered with the subfamily Galerucinae in both analyses, forming branches with high node support values. Furthermore, *O. decempunctatus* and *G. depressa* were found to be sister groups with high support in both maximum likelihood and Bayesian analyses. This study provides important foundational information for further research on the mitochondrial genomes of Chrysomelidae.

## Graphical abstract

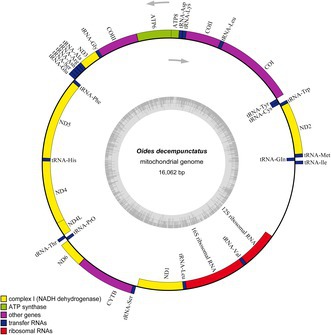

## 1 Introduction

*Oides decempunctatus* is a beetle species in the order Coleoptera and the family Chrysomelidae, found in several regions of China—including Hainan, Guangxi, Sichuan, and Guizhou—as well as in Japan, South Korea, and Vietnam. Its hosts include *Vitis vinifera* , *Vitis flexuosa*, and *Hedera nepalensis*, among other woody climbing plants. Both larvae and adults primarily pose a threat to the buds and leaves of these plants (Li et al. 2011). Their feeding behavior can result in holes or notches on the leaves, and in severe cases, the entire leaf may be consumed, leaving only the vein structure behind. This feeding habit can cause severe damage to the buds and leaves of grapevines, thereby affecting the plant's normal growth and fruit yield. In recent years, with the increasing damage caused by *O. decempunctatus* to plants in the grape family, scholars' interest in this insect has grown significantly.

Mitochondria are semi‐autonomous organelles within eukaryotic cells that possess their own genetic system. They play a crucial role in key biochemical processes such as oxidative phosphorylation and are the primary site of cellular energy metabolism. They contain their own genetic material, exhibit a maternal inheritance pattern, and typically have a low recombination rate and highly conserved gene orders. These characteristics make mitochondrial genomes a valuable resource for population genetics, phylogenetic analysis, and molecular evolutionary studies (Driscoll et al. 2015; Feng et al. 2015; Nelson et al. 2012; Wolstenholme 1992). In recent years, with next‐generation sequencing technology, mitochondrial genome sequencing has become more efficient and cost‐effective, providing a wealth of molecular markers for phylogenetic analysis (Fagua et al. 2018; Feng et al. 2015). In the phylogenetic studies of arthropods, the analysis of mitochondrial sequences is an important tool for tracing their evolutionary radiation pathways and dynamic hybridization events (Moore et al. 2013; Rubinoff et al. 2010; Wang et al. 2017).

The application of mitochondrial genomes in animal phylogenetic inference is very extensive, covering various levels from higher to lower taxonomic ranks (Li et al. 2016; Zheng et al. 2018), including studies on the phylogenetic relationships of beetles at the tribal (Nie et al. 2018), subfamily (Nie, Andújar, et al. 2020; Nie, Vogler, et al. 2020) and family (Nie, Andújar, et al. 2020; Nie, Vogler, et al. 2020) levels. Despite the significant value of mitochondrial genomes in insect phylogenetic research, studies on the mitochondrial genomes of specific species, such as *O. decempunctatus*, are still relatively scarce. This study aims to fill this gap by determining the complete mitochondrial genome sequence of *O. decempunctatus* and comparing it with the mitochondrial genome sequences of 24 species of Chrysomelidae insects available in the GenBank database. Furthermore, this study integrated the gene sequences of the mitochondrial genomes of beetles from GenBank, conducted comparative analysis, and constructed a phylogenetic tree. This not only enriches the database resources of Chrysomelidae mitochondrial genomes but also provides important molecular evidence for the phylogenetic relationships and taxonomic studies of insects within this family.

## 2 Material and Methods

### 2.1 Sample Collection and DNA Extraction

Specimens of *O. decempunctatus* were collected in July 2024 from Nanchong City, Sichuan Province, China (geographic coordinates: 30.816997° N, 106.054208° E, altitude 283 m; Sample collection was conducted in accordance with applicable regulations, and no specific permission was required from local legal authorities for this study). These specimens are preserved in the Forest Protection Laboratory at the College of Life Sciences, China West Normal University. For long‐term preservation, the specimens were soaked in 95% ethanol and stored under conditions of −24°C, and then transferred to the specimen collection room at China West Normal University for storage. Following the manufacturer's instructions, total genomic DNA was extracted from the muscle tissue of individual specimens using the Ezup Chromatography Column Animal Genome DNA Purification Kit (from Shanghai, China). The extraction process adhered to the steps outlined in the kit's manual to ensure the quality and purity of the DNA. The extracted DNA was used for subsequent sequencing work and stored at −24°C to maintain its stability and prevent degradation.

### 2.2 DNA Sequencing, Mitogenome Assembly and Annotation

Beijing Aosen Gene Technology Co. Ltd. (located in Beijing, China) was responsible for the sequencing and assembly work and has successfully obtained the complete mitogenome sequence of *O. decempunctatus*. After quantifying the whole genome DNA, the whole‐genome shotgun strategy was employed to perform sequencing on the Illumina HiSeq sequencing platform. The sequencing process generated a total of 15,843,570 paired‐end reads of 150 base pairs in length, of which 18,880 sequences were used for the assembly of the mitochondrial genome. The assembly process employed the method described by Hahn et al. (2013). The *Monolepta quadriguttata* mitochondrial genome sequence (KY039102) was selected as the reference sequence for this project. The project ultimately succeeded in assembling the full‐length sequence of the mitochondrial genome, which is 16,062 base pairs in length, with a coverage of 174.8 X. Using Geneious 11.0.2 software (Greiner et al. 2019), the protein‐coding genes (PCGs) and rRNA genes were primarily identified by comparing with existing species mitochondrial genome sequences in the GenBank database. The species included are *M. quadriguttata* (KY039102), *Macrohaltica subplicata* (MG021083), and *Altica fragariae* (MH477600). The tRNA genes were carried out by predicting through the tRNAScan‐SE server v 1.21 (Lowe and Eddy 1997) and the MITOS WebServer (Bernt et al. 2013). The relative Synonymous Codon Usage (RSCU) values were calculated using CodonW software (https://codonw.sourceforge.net/), which is a widely used tool for analyzing codon usage bias. Additionally, compositional bias analysis was performed based on the following formulas to calculate AT skew and GC skew: AT skew = (A−T)/(A + T) and GC skew = (G−C)/(G + C) (Perna and Kocher 1995).

### 2.3 Phylogenetic Analyses

This study covers the complete mitochondrial genome data of 25 species of Chrysomelidae insects, including one newly sequenced species (*O. decempunctatus*). The species involved belong to 18 different genera within the Chrysomelidae family, with *Chrysomela vigintipunctata* and *Chrysomela aeneicollis* selected as outgroups to construct the phylogenetic tree (Table 1). The research is based on phylogenetic analysis using 13 PCGs and two RNA genes. The phylogenetic tree was constructed using PhyloSuite v.1.4.4 software, employing maximum likelihood (ML) and Bayesian inference (BI) methods, along with various best‐fit substitution models. Sequence alignment was performed using MAFFT software, which employs fast Fourier transform (FFT) for efficient multiple sequence alignment. Subsequently, MACSE software was used to optimize the alignment of PCGs, a tool specialized for the alignment of mitochondrial genes. The optimized PCGs sequences were trimmed using Gblocks to remove potentially misleading regions. rRNA sequences were trimmed using trimAl software. The best‐fit substitution models for ML and BI analyses were calculated using ModelFinder v.2.3 software. ML analysis was conducted with 1000 ultrafast bootstrap tests and 1000 SH‐aLRT approximate likelihood ratio tests (SH‐aLRT) to assess node support in the tree. Bayesian analysis was run with two independent chains, each with 1 million generations, sampling every 100 generations, and setting a 25% burn‐in to ensure convergence. The final phylogenetic tree was visualized and edited using the iTOL online server (https://itol.embl.de/).

**TABLE 1:** GenBank accession numbers of species used in this study (accession number of the newly sequenced species in bold).

Family	Genus	Species	GenBank number
Chrysomelidae	*Gastrolina*	*Gastrolina depressa*	OR947698.1
		*Gastrolina thoracica*	MF198406.1
	*Plagiodera*	*Plagiodera versicolora*	OR398224.1
	*Leptinotarsa*	*Leptinotarsa decemlineata*	MZ189364.1
	*Chrysolina*	*Chrysolina aeruginosa*	MT826861.1
	*Gastrophysa*	*Gastrophysa atrocyanea*	PQ009943.1
	*Gonioctena*	*Gonioctena intermedia*	MF563962.1
	*Aulacophora*	*Aulacophora indica*	MN162709.1
		*Aulacophora lewisii*	KY039109.1
	*Chaetocnema*	*Chaetocnema pelagica*	MG021087.1
	*Diorhabda*	*Diorhabda carinulata*	MK359257.1
		*Diorhabda carinata*	MK359256.1
	*Galeruca*	*Galeruca daurica*	KR025478.1
	*Luperomorpha*	*Luperomorpha xanthodera*	ON631248.1
	*Oides*	***Oides decempunctatus***	**PQ100713.1**
	*Monolepta*	*Monolepta wilcoxi*	OR582726.1
		*Monolepta bicavipennis*	OR582724.1
		*Monolepta cavipennis*	OR582725.1
		*Monolepta signata*	OM867791.1
	*Podagricomela*	*Podagricomela nigricollis*	MH325078.1
	*Ophraella*	*Ophraella communa*	KY039100.1
	*Argopistes*	*Argopistes tsekooni*	MK060107.1
	*Lema*	*Lema decempunctata*	ON257014.1
Out groups	*Chrysomela*	*Chrysomela vigintipunctata*	MT084795.1
		*Chrysomela aeneicollis*	OP787486.1

## 3 Results

### 3.1 Mitogenome Organization and Composition

**FIGURE 1:**
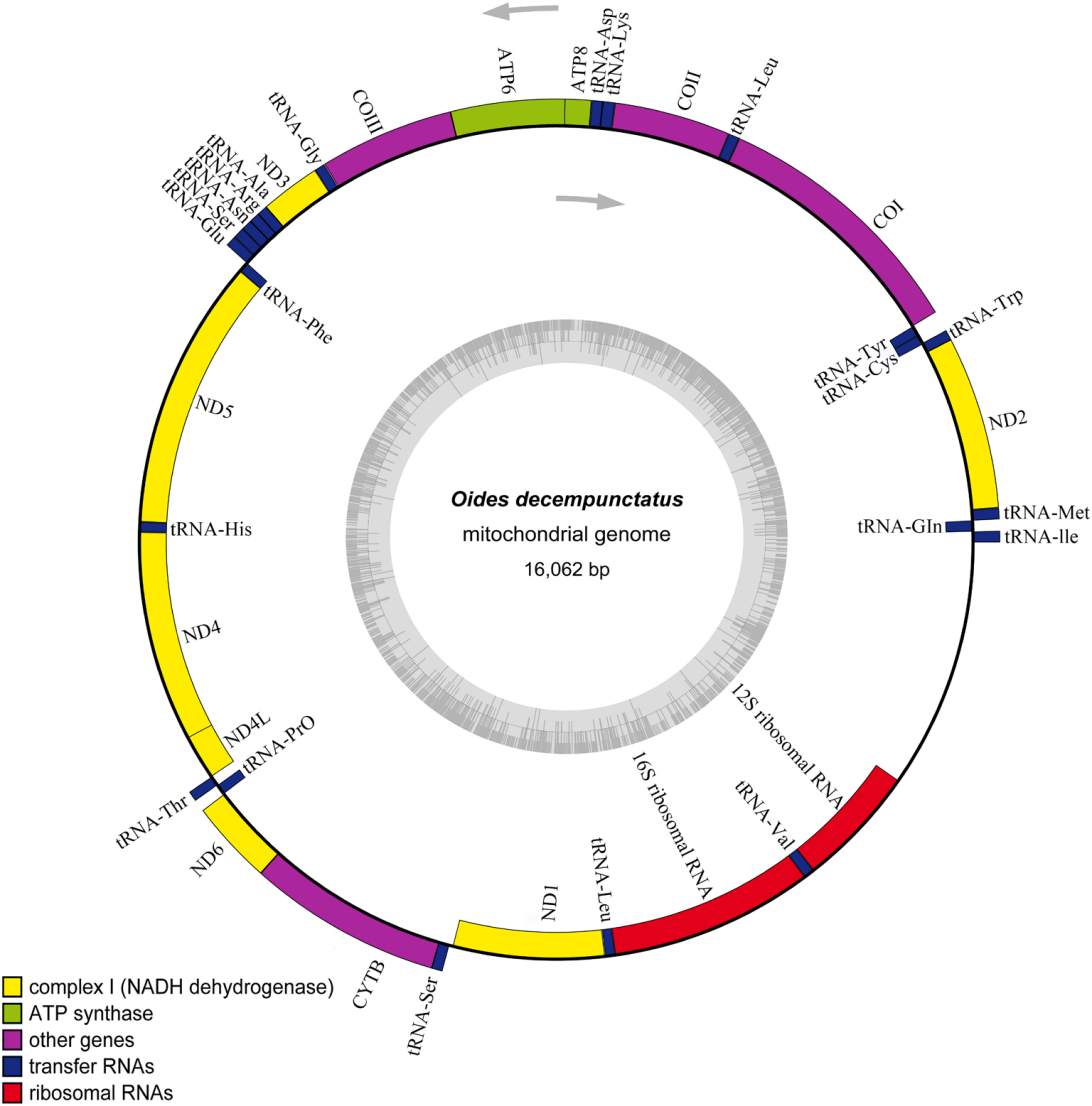
Mitogenome map of *Oides decempunctatus*. ND1, ND2, ND3, ND4, ND4L, ND5, and ND6 are in yellow. COI, COII, and COIII are in magenta. ATP6 and ATP8 are in green. CYTB is in pink. rrnl and rns are in red. All tRNAs are in dark blue and the control region is in white.

The mitochondrial genome of *O. decempunctatus* has been submitted to GenBank under the accession number PQ100713.1. It is 16,062 base pairs in length and exhibits the typical circular, double‐stranded structure characteristic of mitochondrial DNA. The mitochondrial genome comprises 22 transfer RNA (tRNA) genes, two ribosomal RNA (rRNA) genes (12S rRNA and 16S rRNA), 13 PCGs, and a non‐coding control region (Table 2). The gene arrangement in the newly sequenced mitochondrial genome conforms to the ancestral pattern observed in arthropods (Braband et al. 2010). Among these genes, eight tRNA genes (for amino acids Gln, Cys, Tyr, Phe, His, Pro, Leu1, and Val), four PCGs (ND5, ND4, ND4L, and ND1), and the two rRNA genes (12S rRNA and 16S rRNA) are located on the N‐strand of the mitochondrial genome. The remaining 14 tRNA genes (for amino acids Ile, Met, Trp, Leu2, Lys, Asp, Gly, Ala, Arg, Asn, Ser1, Glu, Thr, and Ser2) and nine PCGs (ND2, COI, COII, ATP8, ATP6, COIII, ND3, ND6, and CYTB) are situated on the J‐strand (Figure 1). The nucleotide composition analysis of the mitochondrial genome reveals a high abundance of adenine (A) and thymine (T), accounting for 41.79% and 37.67%, respectively, while cytosine (C) and guanine (G) are present at lower levels, 11.99% and 8.54%, respectively. The total A + T content reaches 79.47%, indicating a strong AT bias, which is consistent with the typical characteristics of insect mitochondrial genomes. Additionally, the AT skew ((A–T)/(A + T)) is 0.05, and the GC skew ((G–C)/(G + C)) is −0.17, further supporting the observation that A is more prevalent than T and G is less abundant than C.

**TABLE 2:** Base composition in different regions of the mitochondrial genome of *Oides decempunctatus*.

Region	Length (bp)	A%	T%	C%	G%	A + T%	AT‐skew	GC‐skew
ND1	942	28.98	48.98	7.96	14.12	77.92	−0.26	0.28
ND2	1014	36.19	42.5	11.83	9.47	78.7	−0.08	−0.11
ND3	354	37.57	44.92	9.32	8.19	82.49	−0.09	−0.06
ND4	1315	30.42	50.65	7.53	11.41	81.06	−0.25	0.20
ND4L	282	30.14	52.13	6.38	11.35	82.27	−0.27	0.28
ND5	1687	31.83	49.44	7.59	11.14	81.27	−0.22	0.19
ND6	495	41.62	42.42	9.9	6.06	84.04	−0.01	−0.24
ATP6	672	35.86	41.82	13.24	9.08	77.68	−0.08	−0.19
ATP8	156	44.87	48.08	5.13	1.92	92.95	−0.03	−0.45
COI	1534	31.16	39.63	15.06	14.15	70.8	−0.12	−0.03
COII	682	36.36	38.71	13.93	11	75.07	−0.03	−0.12
COIII	789	34.35	40.18	13.43	12.04	74.52	−0.08	−0.05
CYTB	1137	34.83	39.84	14.42	10.91	74.67	−0.07	−0.14
Rrnl	1269	38.69	44.52	5.52	11.27	83.22	−0.07	0.34
Rrns	762	38.98	45.28	4.72	11.02	84.26	−0.07	0.40
rRANs	2031	38.8	44.81	5.22	11.18	83.6	−0.07	0.36
tRNAs	1428	41.04	39.64	8.12	11.2	80.67	0.02	0.16
13PCGs	11,059	33.5	44.36	10.99	11.15	77.86	−0.14	0.01
A + T‐rich region	1554	43.5	40.8	9.52	6.18	84.3	0.03	−0.21
Whole Genome	16,062	41.79	37.67	11.99	8.54	79.47	0.05	−0.17

### 3.2 PCGs And Codon Usage

**FIGURE 2:**
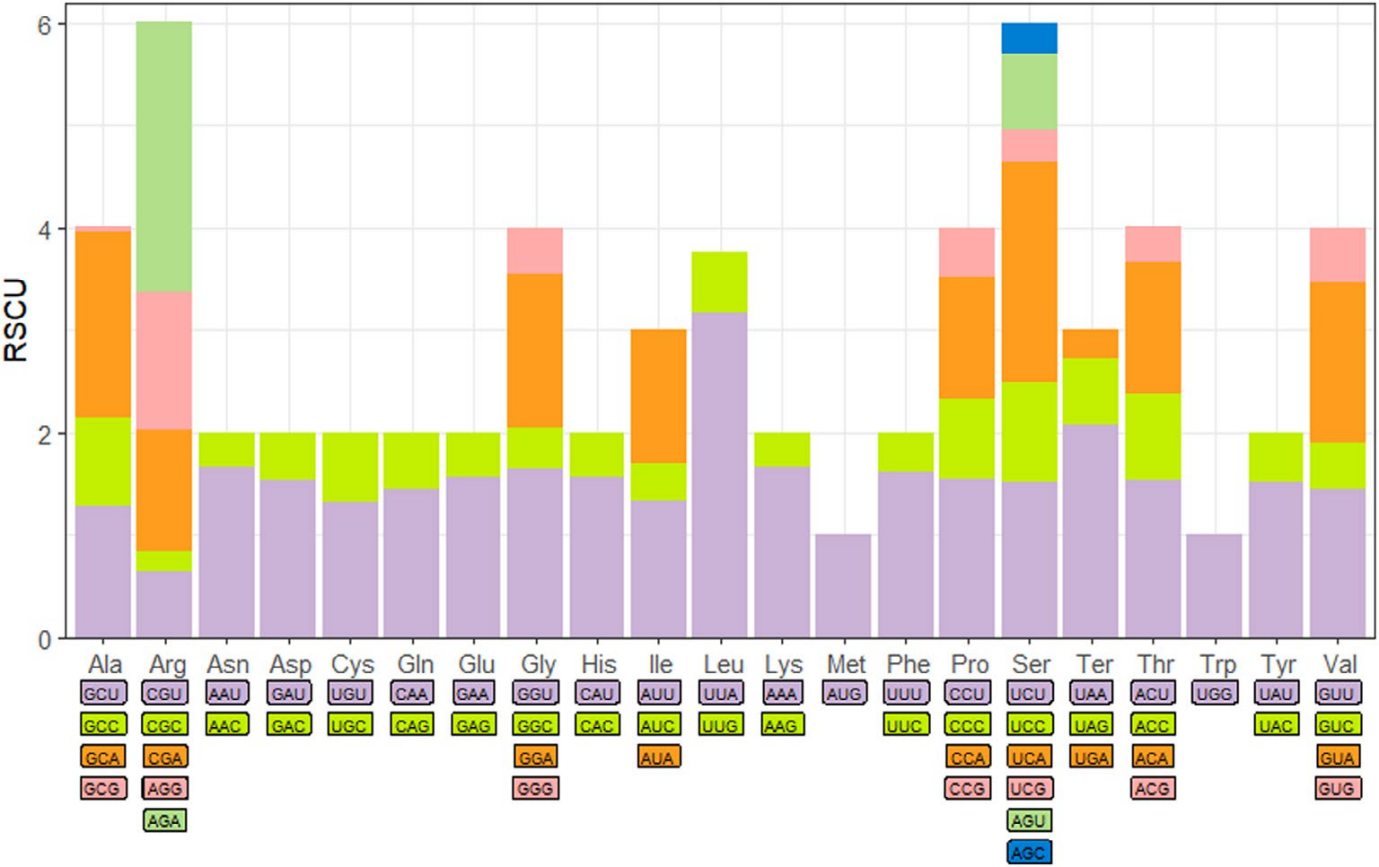
Relative synonymous codon usage (RSCU) of the obtained mitogenome. RSCU value is represented on the *y*‐axis, and the *Oides decempunctatus* codons of their respective amino acids are represented on the *x*‐axis.

The mitochondrial genome of *O. decempunctatus* contains 13 PCGs with a total length of 11,059 base pairs, which accounts for approximately 68.85% of the entire mitochondrial genome. Among these 13 PCGs, four genes (ND5, ND4, ND4L, and ND1) are located on the N strand, while the remaining nine genes (ND2, COI, COII, ATP8, ATP6, COIII, ND3, ND6, and CYTB) are found on the J strand. Of these genes, ND5 is the longest gene, with a length of 1687 base pairs, whereas ATP8 is the shortest, at 156 base pairs. The start codons of mitochondrial genes generally follow the ATN pattern (where N represents A, G, C, or T), with some exceptions. For example, ND1 initiates with the start codon TTG, while the start codon for COI remains undetermined. Regarding stop codons, nine PCGs terminate with the typical TAN codons (TAA or TAG), with TAA and TAG acting as stop signals for different genes. The remaining four PCGs (COI, COII, ND5, and ND4) use a single thymine (T) as an incomplete stop codon (Table 3). It is widely believed that incomplete codon structures in insects and other invertebrates indicate the termination of protein translation (Cheng et al. 2016). Analyzing codon usage in the mitochondrial genome can offer valuable insights into mitochondrial genome replication and transcription mechanisms (Wei et al. 2017). By calculating the RSCU values, we can understand which codons are most prevalent in this genome. In this study, UUA, AGA, UCA, and UAA are the four most frequently used codons (Figure 2).

**TABLE 3:** Characteristics of *Oides decempunctatus* mitochondrial genome. J and N represent the positive and negative chains of the mitochondrial genome, respectively.

Gene	Strand	Region	Length (bp)	Start codon	Stop codon	Anticodon
tRNA‐Ile	J	1–67	67			GAU
tRNA‐Gln	N	67–135	69			UUG
tRNA‐Met	J	134–200	67			CAU
ND2	J	201–1214	1014	ATT	TAA	
tRNA‐Trp	J	1213–1275	63			UCA
tRNA‐Cys	N	1268–1328	61			GCA
tRNA‐Tyr	N	1329–1393	65			GUA
COI	J	1395–2928	1534	—	T	
tRNA‐Leu2	J	2929–2993	65			UAA
COII	J	2994–3675	682	ATT	T	
tRNA‐Lys	J	3676–3746	71			UUU
tRNA‐Asp	J	3746–3811	66			GUC
ATP8	J	3812–3967	156	ATA	TAA	
ATP6	J	3961–4632	672	ATG	TAA	
COIII	J	4632–5420	789	ATG	TAA	
tRNA‐Gly	J	5423–5484	62			UCC
ND3	J	5485–5838	354	ATC	TAG	
tRNA‐Ala	J	5837–5899	63			UGC
tRNA‐Arg	J	5899–5959	61			UCG
tRNA‐Asn	J	5959–6022	64			GUU
tRNA‐Ser1	J	6023–6088	66			CUA
tRNA‐Glu	J	6089–6153	65			UUC
tRNA‐Phe	N	6152–6216	65			GAA
ND5	N	6217–7903	1687	ATT	T	
tRNA‐His	N	7904–7966	63			GUG
ND4	N	7967–9281	1315	ATT	T	
ND4L	N	9275–9556	282	ATG	TAA	
tRNA‐Thr	J	9558–9620	63			UGU
tRNA‐Pro	N	9621–9686	66			UGG
ND6	J	9689–10,183	495	ATA	TAA	
CYTB	J	10,183–11,319	1137	ATG	TAG	
tRNA‐Ser2	J	11,318–11,383	66			UGA
ND1	N	11,405–12,346	942	TTG	TAG	
tRNA‐Leu1	N	12,348–12,410	63			UAG
rRNl	N	12,411–13,679	1269			
tRNA‐Val	N	13,680–13,746	67			UAC
rRNs	N	13,747–14,508	762			
Control region	J	14,509–16,062	1554			

### 3.3 tRNA Genes, rRNA Genes and A + T‐Rich Region

In the mitochondrial genome of *O. decempunctatus*, 22 tRNA genes have been identified, with a total length of 1428 base pairs. The lengths of the individual tRNA genes range from 61 to 71 base pairs. Among them, tRNA‐Lys is the longest, while tRNA‐Cys and tRNA‐Arg are the shortest. The nucleotide composition of the tRNA genes is characterized by a high proportion of adenine (A) and thymine (T), at 41.04% and 39.64%, respectively, while cytosine (C) and guanine (G) are less abundant, at 8.12% and 11.20%. The AT skew and GC skew are 0.02 and 0.16, respectively, indicating a slight enrichment of A over T and a relatively balanced distribution of C and G.

Among the 22 tRNA genes, 8 are located on the N strand of the mitochondrial genome, while the remaining 14 are on the J strand. The secondary structures of these tRNA genes were predicted using the tRNAScan‐SE server v.1.21 (Figure S1). All tRNA genes, with the exception of tRNA‐Ser1—which lacks the dihydrouridine arm—exhibit the typical cloverleaf secondary structure, consistent with findings from previous studies on mitochondrial tRNA structures in other insects (van Staden Michaela et al. 2022).

Additionally, the lengths of the 16S rRNA and 12S rRNA genes are 1269 base pairs and 762 base pairs, respectively. The 16S rRNA gene is located between tRNA‐Leu and tRNA‐Val, with a nucleotide composition of A 38.69%, T 44.52%, C 5.52%, and G 11.27%. The 12S rRNA gene is located between tRNA‐Val and the A + T‐rich region, with a nucleotide composition of 38.98% A, 45.28% T, 4.72% C, and 11.02% G. For both rRNA genes, the AT skew and GC skew are −0.07 and 0.36, respectively, which are consistent with the compositional characteristics observed in rRNA genes of other Chrysomelidae insects.

The control region of the *O. decempunctatus* mitochondrial genome, which is A + T‐rich, is located between the trnI and rrnS genes and spans 1554 base pairs. The A + T content in this region is 84.3%, with an AT skew of 0.03 and a GC skew of −0.21, suggesting a potential role in the regulation of mitochondrial genome replication and transcription.

### 3.4 Phylogenetic Analysis

**FIGURE 3:**
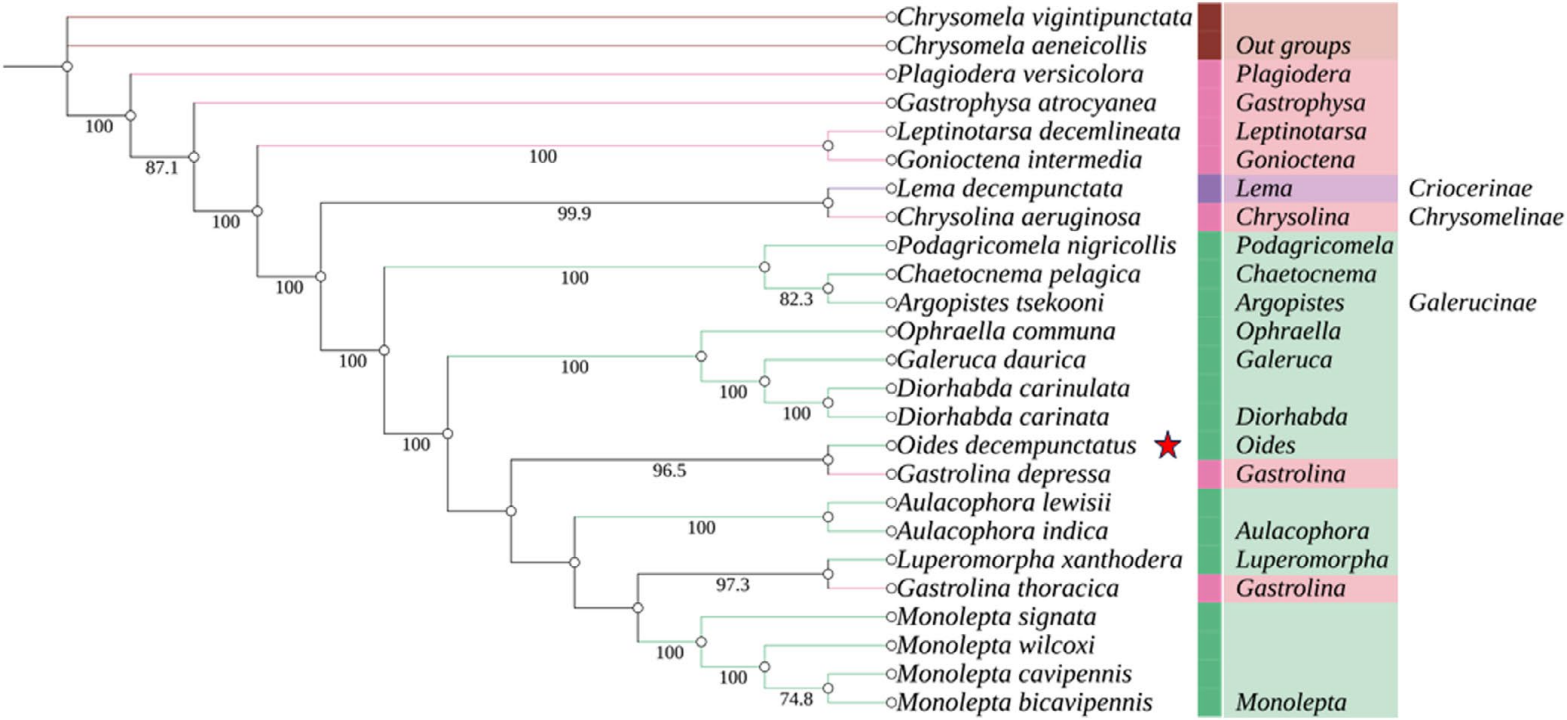
Based on the maximum likelihood analysis of 13 protein‐coding genes (PCGs) and 2 rRNA gene sequences, the phylogenetic relationships of 23 species of leaf beetles and their outgroups (*Chrysomela vigintipunctata* and *Chrysomela aeneicollis* ) were inferred. Values less than 70 are not displayed.

**FIGURE 4:**
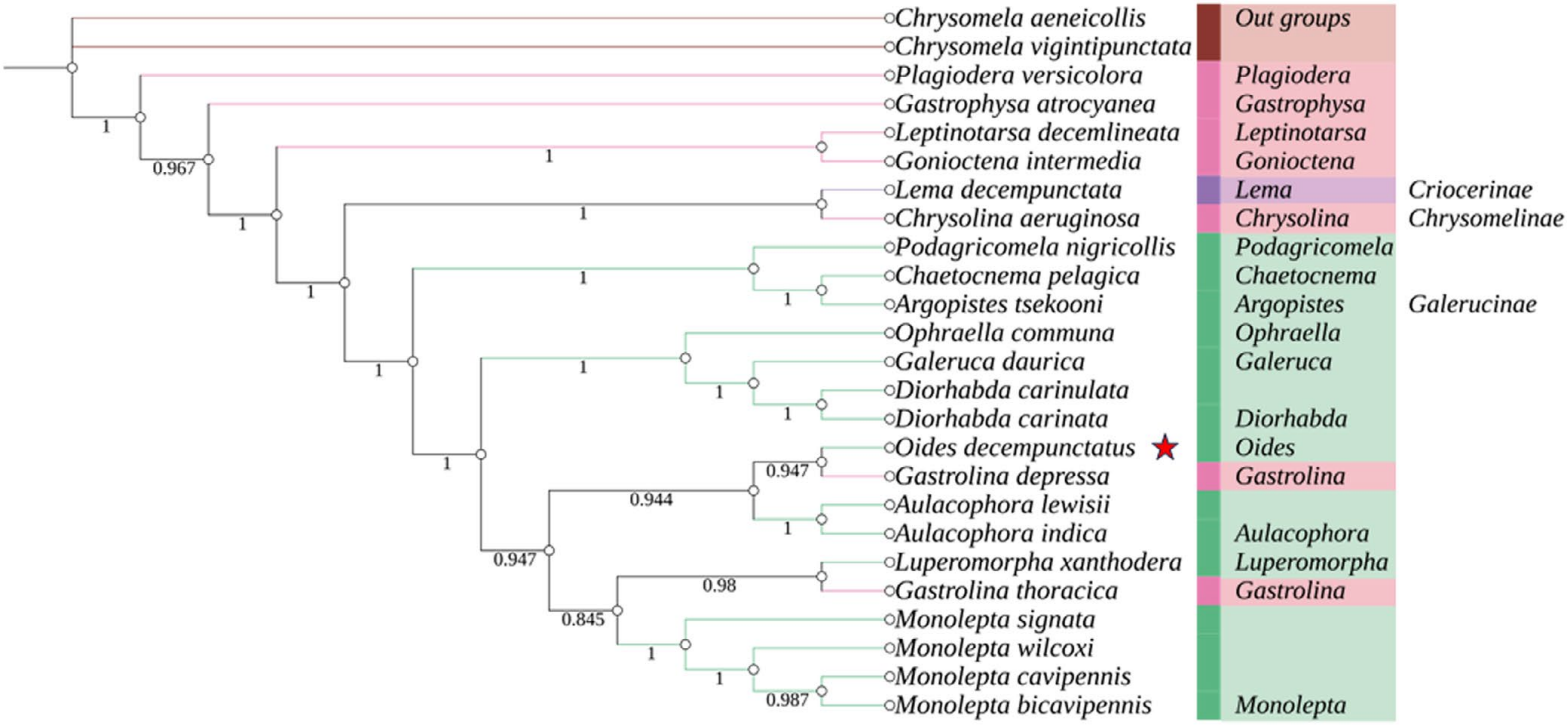
Based on the Bayesian Inference analysis of 13 protein‐coding genes (PCGs) and 2 rRNA gene sequences, the phylogenetic relationships of 23 species of leaf beetles and their outgroups (*Chrysomela vigintipunctata* and *Chrysomela aeneicollis* ) were inferred. Numbers on branches are posterior probabilities.

In insect taxonomy and phylogenetic research, molecular systematics has proven to be a powerful tool for resolving previously ambiguous taxonomic and phylogenetic relationships within the class Insecta. In this study, sequences from 13 PCGs and 2 RNA genes were used to construct a phylogenetic tree of the family Chrysomelidae, employing both ML and BI methods. Despite using different methods and data matrices for tree construction, the phylogenetic trees generated by both ML and BI analyses showed a high level of congruence (Figure 3 and Figure 4). Both approaches provide strong support for the monophyly of Chrysomelidae, indicating that all species within this family form a well‐supported and unified evolutionary clade. This finding suggests that all members of Chrysomelidae share a common ancestor and have diverged into a distinct evolutionary lineage, providing new insights into the evolutionary relationships among species within the family.

However, the phylogenetic analysis also revealed several unexpected evolutionary relationships. For instance, *Lema decempunctata*, a member of the subfamily Criocerinae, clustered with *Chrysolina aeruginosa* from the subfamily Chrysomelinae. In contrast, *Gastrolina depressa* and *Gastrolina thoracica*, also belonging to Chrysomelinae, grouped with species from the subfamily Galerucinae, forming a distinct clade supported by high node values. This finding suggests that *G. depressa* and *G. thoracica* are more closely related to species in Galerucinae than to other members of Chrysomelinae, indicating that current subfamily‐level classifications within Chrysomelidae may not consistently represent monophyletic groups (Lin et al. 2024). Notably, *O. decempunctatus* and *G. depressa* were identified as sister taxa, a relationship strongly supported by both ML and BI analyses. This indicates a close evolutionary relationship between the two species, with robust statistical support for this inference.

## 4 Discussion

This study successfully sequenced and assembled the complete mitochondrial genome of *O. decempunctatus*, a species within the subfamily Galerucinae. Comprehensive annotation and analysis revealed genomic features that enrich the molecular database for Chrysomelidae and provide valuable insights into the genetic diversity of this family.

The mitochondrial genome of *O. decempunctatus* consists of 13 PCGs, 22 tRNA genes, two rRNA genes, and a control region. Notably, the control region is 1554 bp long with an A + T content of 84.3%, contributing to the relatively large genome size compared to other Chrysomelidae species. Most tRNA genes conform to the typical cloverleaf secondary structure, except for tRNA‐Ser1, which lacks the dihydrouridine arm—a feature observed in other insects as well (Liao et al. 2010; Wolstenholme 1992). These findings align with previous observations in insect mitochondrial genomics.

The start and stop codons used by the PCGs are largely consistent with patterns reported in other Chrysomelidae insects. While most PCGs initiate with standard ATN codons, the ND1 gene uniquely starts with TTG. Several genes terminate with incomplete stop codons (single T), which are presumed to be completed via post‐transcriptional polyadenylation (Boore 1999; Coates et al. 2005).

Phylogenetic analyses were conducted using the sequences of 13 PCGs and two rRNA genes from 24 Chrysomelidae species and two outgroup species (*Callosobruchus vigintipunctatus* and *Callosobruchus aeneicollis*), employing both ML and BI methods. Despite minor differences in topology, both methods yielded highly congruent trees, strongly supporting the monophyly of Chrysomelidae. Node support values were generally higher in BI trees, consistent with prior studies (Yuan et al. 2016).

Importantly, *O. decempunctatus* was found to be closely related to *Gastrolina depressa*, forming a sister group relationship with strong support in both ML and BI trees. Unexpected relationships were also observed: *Lema decempunctata* (Criocerinae) clustered with *Chrysolina aeruginosa* (Chrysomelinae), and *G. depressa* and *G. thoracica* (Chrysomelinae) grouped with members of Galerucinae. These results differ from Wang and Tang (2018) but are consistent with Lin et al. (2024), indicating that traditional subfamily classifications may not reflect true evolutionary relationships.

These findings demonstrate the utility of mitochondrial genome data for resolving complex phylogenetic relationships within Chrysomelidae (Heng et al. 2022). However, the exact relationships among subfamilies and tribes remain partially unresolved, emphasizing the need for increased taxon sampling and additional mitochondrial genomes (Lo et al. 2024). For instance, comparative mitogenomic analyses have revealed inconsistencies in traditional subfamily classifications, highlighting the evolutionary complexity within this group (Shengdi et al. 2021). As more complete data become available, we anticipate clearer insights into the evolutionary history and classification of Chrysomelidae insects.

In conclusion, this study contributes a new complete mitochondrial genome to the family Chrysomelidae and provides a well‐supported phylogenetic framework for *O. decempunctatus*. These results not only enhance our understanding of the molecular evolution of Chrysomelidae but also lay a solid foundation for future phylogenetic and taxonomic research.

## Author Contributions

**Honghua Zhang:** formal analysis (equal), methodology (equal), software (equal), writing – original draft (equal). **Junhao Wu:** methodology (equal), writing – review and editing (equal). **Zhiqian Liu:** investigation (equal). **Xinju Wei:** data curation (equal). **Zhihang Zhuo:** conceptualization (equal), supervision (equal), writing – review and editing (equal).

## Conflicts of Interest

The authors declare no conflicts of interest.

## Supporting information


Figure S1.

## Data Availability

The data supporting the results are available in a public repository at: https://doi.org/10.6084/m9.figshare.27002662.v1.
