# Supplementary figures and images for: Complete Mitochondrial Genome and Its Phylogenetic Analysis of Oides decempunctatus (Coleoptera: Chrysomelidae)
Source: Ecol Evol. 2025 Jul 15;15(7):e71819. doi: 10.1002/ece3.71819 (PMC12263182; doi:10.1002/ece3.71819)

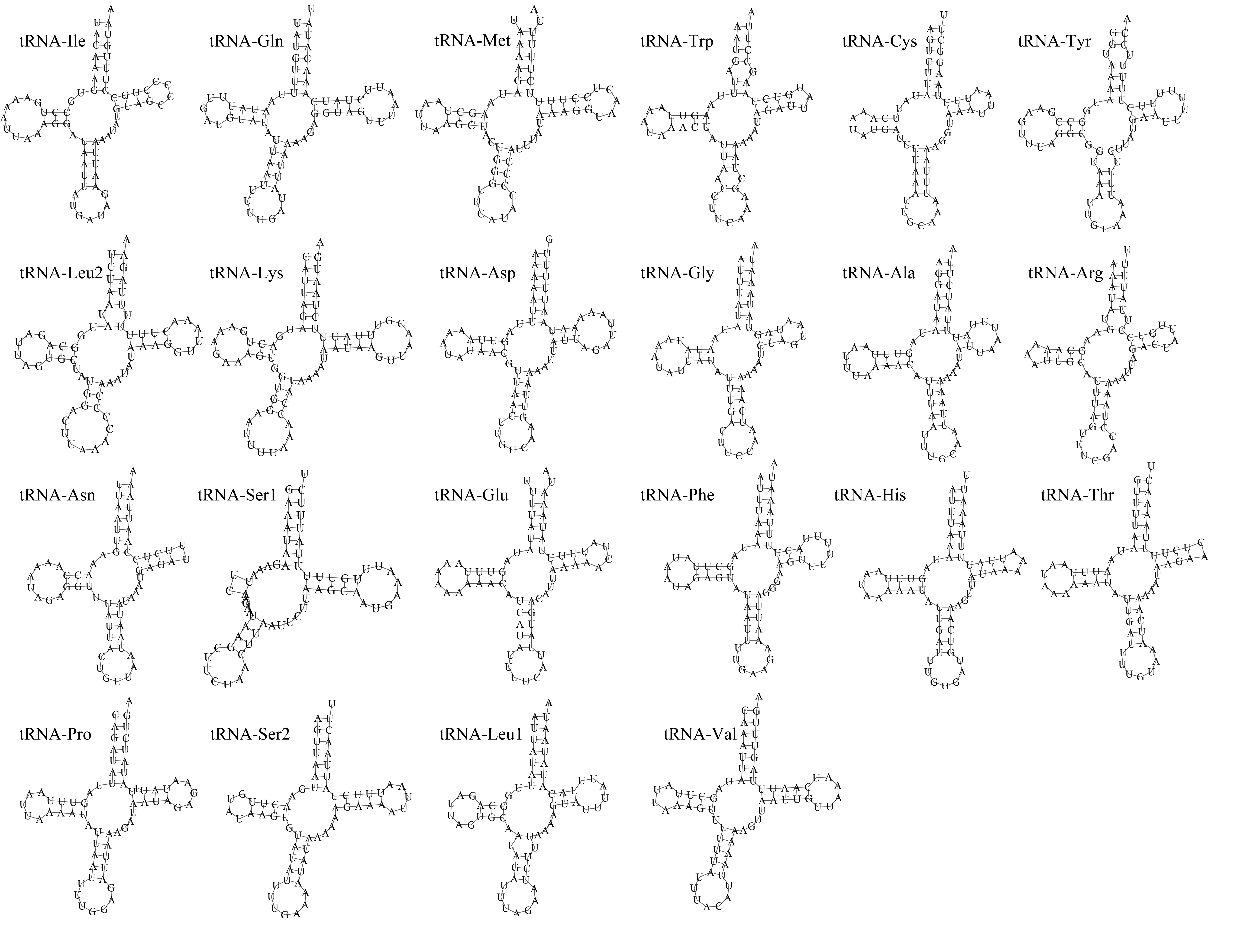

Supplement: Supplementary file 1 — Figure S1. [file ECE3-15-e71819-s001.tif]
